# Introducing payment for performance in the health sector of Tanzania- the policy process

**DOI:** 10.1186/s12992-015-0125-9

**Published:** 2015-09-02

**Authors:** Victor Chimhutu, Marit Tjomsland, Nils Gunnar Songstad, Mwifadhi Mrisho, Karen Marie Moland

**Affiliations:** Department of Health Promotion and Development, University of Bergen, P.O Box 7807, 5020 Bergen, Norway; Faculty of Social Sciences, University of Bergen, P.O Box 7802, 5020 Bergen, Norway; Ifakara Health Institute, P.O Box 78373, Dar es Salaam, Tanzania; Centre for International Health, University of Bergen, P.O Box 7804, 5020 Bergen, Norway

**Keywords:** Payment for performance (P4P), Results-based financing (RBF), Health systems, Low-income contexts, Partnership, Maternal and child health, Health worker motivation, Tanzania

## Abstract

**Background:**

Prompted by the need to achieve progress in health outcomes, payment for performance (P4P) schemes are becoming popular policy options in the health systems in many low income countries. This paper describes the policy process behind the introduction of a payment for performance scheme in the health sector of Tanzania illuminating in particular the interests of and roles played by the Government of Norway, the Government of Tanzania and the other development partners.

**Methods:**

The study employed a qualitative research design using in-depth interviews (IDIs), observations and document reviews. Thirteen IDIs with key-informants representing the views of ten donor agencies and government departments influential in the process of introducing the P4P scheme in Tanzania were conducted in Dar es Salaam, Tanzania and Oslo, Norway. Data was collected on the main trends and thematic priorities in development aid policy, countries and actors perceived to be proponents and opponents to the P4P scheme, and P4P agenda setting in Tanzania.

**Results:**

The initial introduction of P4P in the health sector of Tanzania was controversial. The actors involved including the bilateral donors in the Health Basket Fund, the World Bank, the Tanzanian Government and high level politicians outside the Health Basket Fund fought for their values and interests and formed alliances that shifted in the course of the process. The process was characterized by high political pressure, conflicts, changing alliances, and, as it evolved, consensus building.

**Conclusion:**

The P4P policy process was highly political with external actors playing a significant role in influencing the agenda in Tanzania, leaving less space for the Government of Tanzania to provide leadership in the process. Norway in particular, took a leading role in setting the agenda. The process of introducing P4P became long and frustrating causing mistrust among partners in the Health Basket Fund.

**Electronic supplementary material:**

The online version of this article (doi:10.1186/s12992-015-0125-9) contains supplementary material, which is available to authorized users.

## Background

In the last decade, expenditure on health has increased in many low income countries, but this increase is not commonly matched by better service delivery [1]. User needs and demands are far from met, and in many countries the health system continues to be plagued by inefficiency due to worker absenteeism and resource leakage, poor quality of care and inequity in access to health services [2]. There is a growing confidence in Payment for Performance (P4P) as a tool to address these problems [2], both among donor- and recipient countries. P4P is defined as payment issued upon achievement of a predetermined performance target [3]. While donor countries see the P4P mechanism as an attempt to improve the efficiency of aid by emphasizing measurable results, recipient countries see P4P schemes as an opportunity to fulfil unmet health needs.

There are a number of arguments for and against the use of provider P4P mechanism in the health sector of low income countries and both these arguments are supported by empirical studies. On one hand, it is argued that P4P represents a powerful motivational tool to improve the way health facilities and individual health workers respond to users. It is also argued that P4P can facilitate the pooling and integration of resources and thus improve efficiency and the potential spill-over effect into the whole public sector in low income countries [2]. A study carried out in Rwanda concluded that P4P can be an effective tool to strengthen the quality and the use of maternal and child health services [4]. On the other hand, there are strong arguments against introducing P4P in the health sector. Through the introduction of monetary incentives, it is argued that P4P is ‘crowding out intrinsic motivation’, undermines motivation among workers who are not part of the P4P scheme and erodes social relations and teamwork through competition and envy [5, 6]. Studies in Rwanda and Tanzania have found negative unintended effects of P4P schemes including gaming and the introduction of adverse sanctions [6–8]. The evidence available on P4P schemes in health care is thus inconclusive and cannot be documented across settings [9].

Despite lack of solid evidence on effectiveness, P4P is gaining political support, and a number of countries in sub-Saharan Africa, including Tanzania, are trying out P4P to accelerate the progress towards Millennium Development Goals (MDG) 4 and 5 to improve child and maternal health. Like other low income countries, Tanzania is facing huge challenges in providing good quality health care to its population, and inadequate funds and lack of human and material resources negatively affect the motivation and performance of health workers [10–12]. Less than half of all deliveries are attended by skilled personnel [13], and the quality of birth care is generally poor [14]. Maternal mortality in Tanzania in 2013 was at 390 maternal deaths per 100,000 live births [15] and neonatal mortality in 2013 was at 21 neonatal deaths per 1000 live births [16].

To improve the quality and the utilization of maternal health services a P4P pilot was introduced in Tanzania in 2011 [17]. The decision making process that led to the introduction of P4P involved many bilateral and international partners with different agendas. It has been observed that inadequate attention has been given to policy development processes in the health sector of low income countries [18]. Attention has been paid to the policy contents, ignoring why and how the reforms are carried out and the actors involved [19]. Our study aims to bridge this gap by investigating the policy process behind the introduction of P4P in maternal and child health in Tanzania illuminating in particular the interests and the roles played by the Norwegian Government, the Tanzanian Government and the other development partners. To situate the study we first present the historical ideological context of governance and the more recent partnership model of governance in Tanzania.

### From self-reliance to good governance

The Arusha Declaration of 1967 was based on the political philosophy of Julius Nyerere, the first President of the United Republic of Tanzania, and emphasized central planning and equitable access to services including health care [20–22]. Nyerere and his vision of a self-reliant post-colonial country attracted a lot of attention and aid from countries all around the world [23]. In the 1980s many low income countries adopted structural adjustment policies (SAPs) promoted by the International Monitory Fund (IMF) and the World Bank as a necessary condition for borrowing money and securing economic growth [22, 23]. SAPs involved the scaling down of the public sector and stimulated private sector growth. Nyerere resisted the pressure from IMF and the World Bank to introduce structural adjustment policies in Tanzania [21, 23, 24], but in the wake of the oil crisis in 1973 and a costly military intervention in Uganda to overthrow Idi Amin in 1978–79, Tanzania was in an economic crisis and in need of more aid [23, 25]. Nyerere left office in 1985, paving way for a new administration [23, 24] led by president Ali Hassan Mwinyi, who had no option but to agree to the demands of IMF and the World Bank.

A World Bank report of 1989 [26] defined the development challenges in Africa as a crisis of governments’ inability to manage national affairs, or of *governance*, and argued for a new development paradigm based on *good governance* [27]. Good governance is defined as a governing system “epitomized by predictable, open and enlightened policy making; a bureaucracy imbued with a professional ethos; an executive arm of government accountable for its actions; and a strong civil society participating in public affairs; and all behaving under the rule of law” [28]. Under the good governance paradigm, the notion of partnership between development partners is central. Tanzania opened up to this new paradigm.

### Partnership in the health sector

The relationship between Tanzania and donor countries has not always been smooth with regard to agenda setting and ownership. In 1995, sour relations between the Government of Tanzania and donors led to the appointment of an advisory group to assess how the development cooperation between the Government of Tanzania and the official donor organizations could be strengthened and improved. The outcome was a report critical to both the donor countries for not giving Tanzania space for ownership and to the Government of Tanzania for not being proactive in providing leadership [29].

About the same time the Government of Tanzania had secured its first World Bank credit in the health sector [29] and in 1999, a sector plan of action was developed with the aim of pooling funds in the health sector. The Health Basket Fund was established as a pooling mechanism aiming to simplify administration and coordination and give more control to Tanzania [30]. These reforms put Tanzania in the driving seat and worked to increase donor confidence in the country [24].

The Health Basket Fund, as an instrument of Tanzanian ownership of all activities in the health sector, was led by the Ministry of Health and Social Welfare and initially involved six donors: Norwegian Agency for Development Cooperation (Norad), Swiss Development Cooperation (SDC), Danish International Development Agency (Danida), Department for International Development (DFID), Irish Aid, and the World Bank. The Netherlands, Canadian International Development Agency (CIDA), the German Development Bank (KfW), UNFPA and UNICEF joined later [30]. In our study, we are particularly interested in founding members of the Health Basket Fund, as they have insight into the full history of the Health Basket Fund and extensive knowledge of the power dynamics and agenda setting in the Health Basket Fund over time.

### Conceptualizing partnerships

Partnerships are often described in binary terms as either ‘instrumental’ or ‘genuine’, or as either ‘strong’ or ‘weak’ [31, 32]. While the rhetoric of partnership tends to emphasise a strong version which involves reciprocity, policy dialogue and meeting commitments, a weak version implies that decision making processes constantly come under the review of donors, undermining the aim of country ownership [33]. Maxwell and Riddell contend that a weak version of partnership is commonly preferred by donors (24). For partnerships to work well, Crawford propose a framework with four factors indicating a genuine partnership: (1) mutual co-operation between multiple constituencies, both internal and external actors, (2) respect for sovereignty and the right of national actors to determine their own policy options, (3) equitable and meaningful relationship, and (4) time and commitment to build and maintain a strong partnership [31].

Donor-government partnership in Tanzania has been termed a ‘contested process’, one which obscure a more ‘covert and insidious’ expression of power by development partners [33]. The introduction of P4P to improve maternal and child health in Tanzania is a case in point and illustrates a power struggle between shifting alliances within the Health Basket Fund and tensions between the interests of donors and the interests of the country as an agent of its own development.

## Methods

### Study context

The study was carried out in 2012 and 2013 in two locations: Dar es Salaam, Tanzania, and Oslo, Norway.

### Data collection and analysis

A qualitative study design was adopted to explore narratives and perceptions surrounding the introduction of P4P in Tanzania. In-depth interviews were conducted, observational activities were carried out and reviews of policy document and other relevant secondary data were conducted. Data was collected by the first author in two phases, October-November 2012 in Oslo and January-June 2013 in Dar es Salaam. Below is an account of the method we used for data collection and how these methods are triangulated in the study.

#### Participation in the meetings on P4P

The first author participated in a number of meetings on P4P in Dar es Salaam in the period of January 2013 to June 2013 both as a participant and as an observer. Two of the meetings were particularly important. The first was a P4P stakeholders meeting, held in January 2013 which gave an overview of the status of P4P in health care in Tanzania and provided an opportunity to identify influential actors in the field. During this meeting initial contacts with potential informants in Dar es Salaam were made. The meeting also contributed to the identification of potential sources of secondary data for the study. The second meeting occurred when the first author was requested by the P4P joint assessment committee, which consisted of Norad, the World Bank and USAID, to assist as a resource person on literature on P4P in Tanzania and other contexts. This role was important for gaining access to and building rapport with central informants in the study.

Overall, the participation in the P4P meetings were important for gathering background information, for refining the research questions, for the identification of potential informants, for the development of the interview questions [34], and for mapping of secondary data sources.

#### Policy documents

Policy documents were of utmost importance to the study, and were used mainly from a realist perspective [34], that is, as a means to understanding the P4P policy and design in the Tanzanian context. Hence, policy documents were essential in providing background information to the study and in defining the questions and trajectories that were pursued in the in-depth interviews. Policy documents central to our study include: *The Pwani region P4P pilot: design document* [17], *Health sector startegic plan III (July 2009-June 2015) Partnership for delivering the MDGs* [35]. *The national road map strategic plan to accelerate reduction of maternal, newborn and child deaths* [36], *Payment for performance strategy 2008–2015* [37], *Implementation guidelines- payment for performance* [38]. These policy documents have been instrumental in uncovering the political frames and in supplementing primary data collected from the representatives of the Ministry of Health and Social Welfare.

#### In-depth interviews (IDIs)

Detailed information on a range of themes related to the introduction of P4P in the health sector in Tanzania was obtained through IDIs conducted with representatives of organizations and agencies identified during participant observation in the meetings and conferences. The questions that were asked in interviews were tailored to suit the perceived roles played by different actors, and this process was aided by the information obtained in policy documents, and during observations. Three interview guides were designed: one for Government officials in Tanzania; one for Norwegian informants, and one for other development partners and stakeholders. Although these three interview guides had different specific (for detailed interview questions refer to additional files 1, 2, and 3), they were all guided by the following general themes: trends and thematic priorities in development aid policy, countries and actors perceived to be proponents of the P4P scheme, and agenda setting in the Tanzanian Health Basket Fund and the introduction of P4P.

The P4P agenda in Tanzania was first introduced into the Health Basket Fund. In choosing the informants for the study, we used purposive sampling following two criteria. To achieve the objective of the study we were interested in the views of members of the Health Basket Fund who were influential during the P4P introduction process by either supporting or questioning the P4P agenda. Through this criterion, we were able to identify the Ministry of Health and Social Welfare of Tanzania, the World Bank, the Norwegian Agency for Development Cooperation (Norad), the Danish International Development Assistance (Danida), the Swiss Agency for Development and Cooperation (SDC), German International Cooperation (GiZ) and Irish Aid. All these members (with the exception of GiZ) were formative members of the Health Basket Fund in 1999. They were selected based on the assumption that they therefore possessed more knowledge on the founding principles of the Health Basket Fund than members that joined at a later stage. Secondly, we were interested in organizations/agencies outside the Health Basket Fund that appeared to be important stakeholders in the P4P agenda setting and the subsequent P4P pilot. Based on this criterion we identified the Clinton Health Access Initiative (CHAI), an organization managing the P4P scheme in Tanzania on behalf of the Ministry of Health and Social Welfare. In addition, we included the Norwegian Embassy in Dar es Salaam, and the Norwegian Ministry of Foreign Affairs, who played an important role in introducing and funding the P4P programme in Tanzania. From these 10 organizations/agencies, a total number of 13 in-depth interviews with key-informants were conducted, 11 of these in Dar es Salaam and two in Oslo. Informant selection in the organizations focused on individuals knowledgeable of the P4P agenda setting and process in Tanzania, and the majority of our informants were representatives of their organizations in the Health Basket Fund. An overview of the interviews conducted is summarized in Table 1.

**Table 1:** Overview of IDIs

Agency/Organization	Interviews
Ministry of Health and Social Welfare- Tanzania	2
World Bank – Tanzania	1
Ministry of Foreign Affairs – Norway	1
The Royal Norwegian Embassy – Tanzania	1
Norad	1
Danida	1
Irish Aid	1
GiZ Tanzania	2
Swiss Agency for Development and Cooperation	1
Clinton Health Access Initiative (CHAI)	2

All interviews were conducted in English and based on informed consent, and all except two were recorded. The two interviews were not recorded due to the preference of the informants. In addition to recording, rapid note taking was used in all interviews. The recorded IDIs were transcribed verbatim and error checked. The analysis of the material started with a review of transcripts which were later imported to NVivo 10 for data management purposes. Qualitative content analysis was undertaken, looking for both manifest and latent content [39]. Coding units were identified and condensed [39]. Sub-themes were developed from the codes and defined into themes that we used in presenting our results.

### Research ethics

Research clearance was granted in Norway through the Norwegian Social Science Data Services and in Tanzania through the Ifakara Institutional Review Board, and the National Institute for Medical Research (NIMR/HQ/R.8a/Vol.IX/1515). Individual consent was sought and obtained free of coercion.

## Results

The introduction of P4P in the health sector in Tanzania was controversial. The actors involved, including the bilateral donors in the Health Basket Fund, the World Bank, the Government of Tanzania and high level politicians outside the Health Basket Fund, fought for their values and interests and formed alliances that shifted in the course of the process. In the following we will describe the process with emphasis on 1) the role of high political pressure, 2) the conflicts and changing alliances in the Health Basket Fund, and 3) consensus building.

### High political pressure

The history of Norwegian development aid to Tanzania goes back to the early years after independence, and Norway remains among the most influential donor countries in Tanzania. The idea of P4P in Tanzania originated from the Norwegian-Tanzanian health sector partnership initiative (NTPI) which was signed in 2007 by the President of Tanzania and the Norwegian Prime Minister. The aim of the partnership was to enhance progress to reach MDGs 4 and 5 using P4P mechanism. As all informants pointed out, this was a top-down process. The P4P agenda was defined by high level politicians both from Tanzania and Norway. The prominent role of high level politicians led to a considerable amount of political pressure to introduce P4P in Tanzania. As one informant in the Norwegian Embassy noted:

[Former] *Prime Minister Stoltenberg of Norway and President Kikwete of Tanzania met in 2007, so as you can see the engagement was at a very high level regarding P4P. After this meeting we were requested to support the health sector* [through P4P] *in Tanzania, but prior to this, the embassy wasn’t really visible in the health sector in Tanzania.* (Staff, Royal Norwegian Embassy, Dar es Salaam)

The strong engagement of high level political actors in the P4P agenda kept the involvement of technical actors in defining and shaping the agenda on a low level. In Norway, the Ministry of Foreign Affairs was said to have bypassed the Norwegian Agency for Development Cooperation (Norad), which provides technical support to the Ministry. Norad was not consulted about the P4P agenda and Norad staff expressed great scepticism to its introduction in Tanzania. As one informant pointed out:

*We were raising some questions around P4P since we had a feeling that the treatment was prescribed before the diagnosis, because they said let’s do P4P in Tanzania. Really, without even looking at what are the barriers to the quality of services, to the delivery of services and so on and so forth, but the recipe was already coming, and we quietly and quickly realized that we cannot maneuver much outside this P4P thinking.* (Official, Norad)

In Tanzania, the consensus among some high level bureaucrats and technical staff in the Ministry of Health and Social Welfare was that the health system was not ready for P4P, as it was perceived as a piecemeal reform. It was pointed out that there was need for a reform that takes a systems approach to the challenges in the health sector of Tanzania, as highlighted by the following quote:

*The primary problem that we are facing in Tanzania is a health system that isn’t working well. If you think of the six building blocks of a health system, all those, including financing, infrastructure, health management information systems, among others, P4P could have worked well if all these blocks were functioning well, so around P4P you need to get the system working well for desired results.* (Official - Ministry of Health and Social Welfare, Tanzania)

Partners in the Health Basket Fund, among them Danida, SDC and Irish Aid, were not happy about how the P4P agenda was introduced. The partners felt that P4P was being pushed from above without adequate evidence showing that such mechanisms work in low income contexts. Officials from these international development agencies interact with officials from the Government of Tanzania regularly; hence there was a common understanding that the P4P agenda was driven by high level politicians. The understanding was that technical staff in the Ministry of Health and Social Welfare in Tanzania was not in a position to oppose or refuse the P4P agenda. As one informant put it:

*The thing is that there is political drive and political push to go for that* [P4P] *and this political push comes from Norway and therefore the government* [of Tanzania] *was not in a position to say no despite that the basic foundations to support P4P, either at health facilities or in the health system were not available.* (Official, Danida)

Our reviews of policy documents showed a marked lack of progress in health outcomes relating to MDG 4 and 5 in Tanzania [35, 36] and there was great pressure on the Government of Tanzania to find a way of improving these health outcomes and reach the international targets in child and maternal health. The need to document better health outcomes stimulated and justified a search for new strategies. As one official expressed:

*People were saying we are not achieving enough and we were mainly concerned that we might not reach targets for health related MDGs, especially goals 4 and 5. Because of this we were thinking of a way to accelerate progress towards these targets.* (Official - Ministry of Health and Social Welfare, Tanzania)

When politicians in Tanzania were searching for ways to make progress in MDG 4 and 5, politicians in Norway were looking for partners willing to use P4P schemes in maternal and child health. One informant recalls how Tanzania was chosen as a potential P4P partner:

*The Norwegian government wanted to go into countries that were really struggling with child mortality as well as infant mortality, and so countries were picked according to that. India was one, and then Pakistan was chosen, and then there was a need for some countries in Africa. Tanzania became the obvious choice, because it is a relatively easy country to work in, in terms of stable political conditions, and also quite strong leadership, with a strong President.* (Official, Norad)

### Conflicts and changing alliances in the health basket fund

The Government of Norway pulled out of the Health Basket Fund in 2002, but re-joined in 2007, presumably for the purpose of financing the P4P scheme in the health sector. The Ministry of Foreign Affairs engaged Norad as the Norwegian partner in the Health Basket Fund with the assignment of introducing the P4P agenda to the Health Basket partners. The move was not positively received by the majority of actors in the Health Basket Fund:

*Norad was just rejoining the health basket at the time when we introduced the P4P agenda. People did not approve of that, especially coming with such an agenda of P4P, some of the donors were totally against it, like the Danes, they were appalled by it both politically and otherwise. Even the World Bank and USAID could not come openly to support us for fear of a backlash. The Dutch were furious, saying we were not serious, calling us names, and saying we were trying to hijack the Health Basket Fund.* (Official, Norad)

Other development partners perceived it as disrespectful to introduce such a highly value-laden and politically charged agenda without broad consultation. Pushing the P4P agenda through the Health Basket Fund was interpreted as going against the values of the partnership, especially the earmarking of funds in the basket. The introduction of the agenda was therefore met with resistance in Health Basket Fund.

In addition to conflicting values, opponents of the P4P agenda pointed to the need to evaluate the feasibility of P4P in the health sector of Tanzania in particular and low income contexts in general. In response, Norad commissioned two evaluations in 2008, whose findings did not support the introduction of P4P scheme in Tanzania. The reports concluded that there was lack of evidence on the effectiveness of P4P [40] and that the health system in Tanzania was not ready for a full scale national P4P scheme [41].

However, preparatory work for a full scale national P4P scheme had already started after the signing of the NTPI. In 2008, the Ministry of Health and Social Welfare produced two policy documents: *the Payment for performance strategy 2008–2015* [37], *Implementation guidelines- payment for performance* [38]. The Government of Tanzania was ready to start the implementation of a full scale national P4P scheme. The Health Basket Fund partners, notably Danida, SDC, and Irish Aid which were and still are among the leading contributors of funds the Health Basket continued to resist the agenda. In addition, the position of the Norwegian partner on the agenda was changing mainly because the results from the evaluations did not support a full scale national P4P scheme. Instead, Norad proposed a P4P pilot in one region. This suggestion was openly supported by some of the members in the Health Basket Fund among them The World Bank and USAID, but was rejected by the Government of Tanzania:

*The government’s position back then was that there were too many pilots in the country and if there was going to be anything it has to go full-scale. A pilot would mean that one district or region would benefit. Tanzania has a strong feeling about equity issues, you know from our history, and because of this the government was determined to go ahead with a full-scale implementation.* (Official - Ministry of Health and Social Welfare, Tanzania)

While acknowledging the lack of adequate conditions to implement a full scale national P4P scheme, the Government of Tanzania was adamant that they do not need perfect conditions to start the scheme, instead they preferred a *“learning by doing-approach”*. In 2009, the Government of Tanzania went ahead attempting the implementing of a full scale national P4P scheme. The Health Basket Fund partners were not happy about this move, including Norad:

*P4P came with pressure such that the government was forced to go full scale with P4P. Yet the system was not ready and it didn’t function and the basket partners said we cannot do it. We pulled out and did not finance that. This was a real blow because it created tensions between basket partners and the government. I really feel sorry. We lost valuable time, energy and confidence in this process.* (Official, Swiss Development Cooperation)

The attempted full scale national P4P scheme did not receive funding from the Health Basket Fund, but a few districts implemented the scheme from 2009 to 2011. With no funding and without a proper Health Management Information Systems in place, the full scale national P4P scheme faced a number of challenges.

The common position against the Government of Tanzania’s full scale national P4P scheme improved relations among donor partners in the Health Basket Fund. Most notably was the open support of the World Bank to the proposal of Norad for a P4P pilot in one region. Being the main funder of the Health Basket Fund, the support of the World Bank was important in redirecting the P4P agenda in Tanzania. The interest and active pursuit of the P4P agenda by the World Bank was not well received by all members in the Health Basket Fund as some were still skeptical to the agenda.

*The World Bank is now putting P4P as a condition for funding the basket. P4P may not be bad as such, but we would expect the Bank to come with a lot of expertise and negotiate with all the partners to get that* [the agenda] *through, but it was not exactly like that. It was discussed with the partners but I am not sure if there was broad consensus on this approach. It was pushed on the Basket and now we have to make the best of it.* (Official, Swiss Development Cooperation)

As the full scale national P4P scheme became increasingly difficult, the Government of Tanzania softened its stance on the scope of the P4P scheme. Together with the World Bank, Norad and USAID plans for a P4P pilot in the Pwani Region were started. In 2011, the Pwani Region pilot was introduced in Tanzania with the aim of scaling up after evaluation. The pilot was a result of constant changes in alliance among members in the Health Basket Fund.

### Building consensus

The potential of scaling-up the P4P pilot meant that a common position regarding the agenda needed consensus among members of the Health Basket Fund. This consensus building process had begun during our data collection period. The Government of Tanzania, the World Bank, Norad, and USAID were leading this process, as the following statement illustrates:

*Things were not very clear when P4P was introduced. People needed more understanding of the design and operations of the program, which was not readily available. Many were skeptical of the design, so Norway, which is one of the members in the Basket, asked if we could look more into the concept and this is how the pilot came about. Now as we do these assessments, we see that more donors are coming in and a taskforce for P4P has been formed by the Ministry and we have partners like USAID, the World Bank and Norway, the Germans. Also the chair of the basketeers* [Irish Aid at the time] *is being co-opted.* (Official, World Bank Tanzania)

In one of the meetings of the Joint Assessment committee comprising USAID, the World Bank and Ministry of Health and Social Welfare, the possibility of inviting other development partners to take part in recently established ‘National P4P Taskforce’ was discussed. Senior level politicians in Tanzania who had strongly argued for a full scale national P4P scheme gradually changed position and increasingly supported the views of their technical staff as shown by the following extract:

*There is an emerging consensus that P4P needs to be viewed within the broader health systems reforms…there is need to make sure facilities receive essential medicines in time, are well equipped and meet minimum staffing standards so that they can perform and deliver quality services*. [Former Minister of Health and Social Welfare, Tanzania [42]]

Opponents of P4P in Tanzania were calling for a whole systems approach in implementing the P4P scheme. The argument was that P4P must not be seen as the panacea to problems facing the health sector of Tanzania; as such the scheme has to be integrated in the existing efforts. With high level political officials in Tanzania calling for a whole systems approach to P4P, the scheme seems to be approaching a large degree of consensus.

## Discussion

In this section we will discuss Norway’s interest in the P4P agenda and partnership contestations, and the role of the government of Tanzania in the P4P agenda setting linking it to the question of ownership.

### Norway’s interest in the P4P agenda and partnership contestations

Our data demonstrates that Norway played an important part in bringing the P4P agenda to Tanzania. Norway’s interest in P4P schemes in the health sector can be traced to Jens Stoltenberg, who was the Norwegian Prime Minister 2000–2001 and 2005–2013. Stoltenberg, an economist by training, supported the idea that saving the lives of children in developing countries is a moral and political imperative which carries economic benefits [43, 44]. In 2007, Stoltenberg launched the *Global Campaign for the Health Millennium Development Goals*, a campaign promoting different initiatives, including P4P schemes, to ensure ‘value for money’ while reaching the most vulnerable groups [45]. In addition to substantial financial support to the UN, global child and maternal health campaigns, and global health initiatives such as GAVI and the Gates Foundation, Norway was and still is engaged in bilateral partnerships with several countries lagging behind in MDGs 4 and 5, including India, Tanzania, Nigeria and Malawi [43, 46].

Through the Norwegian Government’s involvement in health related MDGs, in particular goals 4 and 5, Norway emerged as a prominent player promoting the introduction of innovative financing mechanisms in health and other sectors globally [44, 47, 48]. One should note that there was high interest in the Norwegian policy environment relating to outcomes of MDGs 4 and 5. We will examine this interest using the *agenda-setting circumstances* concept in Grindle and Thomas’s framework on political economy of reform. The framework is used in analyzing policy and organizational reforms in developing countries and its key elements are *environmental context* of reform, the *agenda-setting circumstances* and the *policy characteristics* [18, 49]. We find the *agenda-setting circumstances* adaptable to the global policy agenda setting. According to the framework, the policy *agenda-setting circumstances* can be perceived as either a crisis situation or not [49]. When an *agenda-setting circumstance* is seen as a crisis situation, there is be high political interest and the involvement of policy elites. In such circumstances, there is a sense of urgency to ‘do something’ as political and economic stakes are high for inaction [49]. We argue that the possibility of failing to meet the health related MDGs could be interpreted as a crisis situation, especially by the actors that had been actively supporting them. In the same regard, a perceived crisis situation calls for innovative strategies [49], such as using P4P in the health sector of low income countries to accelerate progress towards MDGs 4 and 5. To this end, the concept of *agenda-setting circumstances* helps to explain the high level political interest in P4P on the Norwegian as well as the Tanzanian side.

The other development partners in Tanzania did not share this strong political interest in P4P. Our data show that these actors in the Health Basket Fund did not approve of the way P4P was introduced in the Health Basket Fund. The introduction was perceived to have been largely politically motivated and not following the principles important in a partnership. To further shed light on this, we apply Crawford’s framework on genuine partnership.

Crawford [31] proposed four principles guiding genuine partnerships. The first principle emphasizes mutual co-operation between actors. Our data suggests that different actors in the partnership had different interests. Alliances were constantly shifting in the Health Basket Fund. It was perceived that Norway ‘pushed’ the P4P agenda before seeking broad consensus and co-operation from all partners. In the eyes of other long-term and major financial contributors in the Health Basket Fund, such as Danida, SDC and Irish Aid, Norway did not respect mutual co-operation, a principle considered to be fundamental for a genuine partnership. This, in our view, contributed to the derailing of the P4P agenda in Tanzania.

The second principle of partnership concerns the sovereignty and right of national actors to make their own policy choices. In the context of the P4P agenda in Tanzania, none of the international donors in the Health Basket Fund observed this important principle. On different occasions the P4P agenda was driven by international actors in the Health Basket Fund and not by the government of Tanzania. Norway and the World Bank took turns in pushing the agenda while the other development partners constantly opposed the P4P agenda, even at times when the Government of Tanzania had resolved to implement the P4P reform. It can be argued that this stance by international actors neutralized the Government of Tanzania’s prerogative to determine its own policy options.

The third principle by Crawford on genuine partnership emphasizes equality. Our data support an image of the Health Basket Fund in Tanzania as a power arena where partners fought to promote their own values and ideologies [31]. This is illustrated in particular by the moment when the World Bank officially showed interest in the P4P agenda which immediately gained momentum. We argue that by being the largest funder in the Health Basket Fund, the World Bank had more bargaining power and clout to impose its worldview. As the process unfolds, we see international actors engaging in power games in leading or resisting the P4P agenda leaving Tanzania as a less equal partner.

The fourth and final principle on a genuine partnership encourages investment of time and commitment in the building of a strong partnership [31]. Norway did not observe this principle. As a former member that re-joined the partnership, Norway was expected to build trust through showing commitment to the basket fund partners over time. When Norway rushed to introduce the P4P agenda despite heavy resistance, Norway sought alliance with the World Bank, a powerful actor in the Health Basket Fund. This alliance over time increased the pro P4P pressure beyond what Norway’s status as single donor country would allow. This was interpreted by other partners as manipulation of a partnership platform supposed to be based on consensus and mutual interest.

Partnership theory tends to see strong partnerships as the ideal or ‘real’ partnerships, while in practice, according to Maxwell and Riddle, donors tend to prefer weak partnerships as it makes it easier to dominate agenda setting [32]. The approach by Norway when introducing P4P to the Tanzanian health sector appears to be a text-book example of this.

It is important to try to understand why the Government of Tanzania was not in control of the P4P agenda, despite a clear interest in it as expressed in the policy documents and by the engagement of high level political actors in the Tanzanian context in setting the P4P agenda.

### The role of the Tanzanian Government in P4P agenda setting

National governments have important roles to play as planners and executors of national welfare systems, and this remains their role also when they engage in partnerships such as the one under scrutiny here. According to partnership theory, the recipient government is supposed to play a pivotal role in priority setting and consensus building among the partners and in assuming ownership to the programs and strategies developed by the partnership [31, 50]. It is therefore important for a proper understanding of the P4P agenda setting to analyze how Tanzania played out its role in the process.

The data presentation above clearly reflects a significant level of ambivalence and indecision on the part of the Tanzanian government. Most informants identified the P4P agenda as owned by the Norwegian government rather than the Tanzanian. Moreover, the attempts actually made by the Tanzanian government to influence the process, such as its initiative to launch a nationally owned P4P scheme, and its later insistence that the program should go to scale, were over-ruled by the other partners in the Health Basket Fund. It is demonstrated that at technical and bureaucratic level, the Government of Tanzania would have preferred a systems approach to health sector challenges and not a standalone P4P initiative. This all suggests that the Tanzanian Government was unable to play the leading role in the process that it was supposed to according to partnership principles. This may have several reasons.

In a recent study of eight African countries’ negotiation capital in aid using the country’s economic, political, ideological and institutional factors as parameters, Tanzania ranked among the weakest states [51]. This strongly suggests that even if it wanted to, the Government of Tanzania’s ability to take a leading role in the negotiations around the introduction of P4P, thereby contributing to making the Health Basket Fund a strong partnership was rather limited. However, and perhaps paradoxically, this may not have been in their interest. As discussed above, donor partners tend to prefer weak partnerships to strong ones because they are more easily managed. Donors, obviously, also control the funding of the partnerships, and may withhold funding or pull out if they find that the partnership moves in a direction they do not approve of, as exemplified by the Tanzanian P4P pilot that the Health Basket Fund refused to fund. Hence, although the partnership model emphasizes the importance of mutual respect, cooperation and sovereignty [31], recipient partners may in fact gain most in terms of funding and goodwill if they give up leadership of the process and ownership of the agenda and follow the lead of the donor partners.

There are several examples in the literature of Tanzania following similar strategies in similar contexts [25, 52] which further suggests that this may in fact be the country’s most preferred and rational approach in development partnerships. This could also illuminate why Tanzania is sometimes referred to as an unofficial ‘darling of the donor community’ [52]. Donor countries tend to find the Government of Tanzania receptive to their ideas and agendas, and respond by maintaining a high level of aid to the country.

## Study limitations

The study lacked the contributions of some of the stakeholders in the Donor Partners Group for Health, which were mentioned in our informants’ narratives such as USAID and the Netherlands, whose views would have enriched our study. In addition, our data could have been enriched if we had managed to include more actors from the Government of Tanzania. However, concerted efforts were made during the course of the fieldwork to get in touch with these organizations with limited and varying success.

## Conclusion

The process of introducing the P4P scheme in Tanzania was fraught with tension, contestations, and mistrust. The donor - government partnership in Tanzania as expressed in the case of the Health Basket Fund, was by and large dominated by donor countries and agencies. This left less space for Tanzania to be proactive as an agent of its own development. The study also demonstrates that while high political interest is important in stimulating reforms, this does not always translate into quick policy decisions.

## Additional files

Additional files 1:
**Interview guide for Norwegian Officials.** (DOC 30 kb) Additional files 2:
**Interview Guide for Tanzanian officials.** (DOC 29 kb) Additional files 3:
**Interview guide for other officials.** (DOC 32 kb)

## References

[1] World health Organization (2014). External aid for health remains insufficient in low income countries.

[2] Meessen B, Soucat A, Sekabaraga C (2011). Performance-based financing: just a donor fad or a catalyst towards comprehensive health-care reform?. Bull World Health Organ.

[3] Eldridge C, Palmer N (2009). Performance-based payment: some reflections on the discourse, evidence and unanswered questions. Health Policy Plan.

[4] Basinga P, Gertler PJ, Binagwalo A, Soucat ALB, Sturdy JR, Vermeersch CMJ (2011). Effect on maternal and child health services in Rwanda of payment to primary health-care providers for performance: an impact evaluation. Lancet.

[5] Magrath P, Nichter M (2012). Payment for performance and the social relations of health care provision: An anthropological perspective. Soc Sci Med.

[6] Kalk A, Paul AF, Grabosch E (2010). ‘Paying for performance’ in Rwanda: does it pay off?. Trop Med Int Health.

[7] Chimhutu V, Lindkvist I, Lange S (2014). When incentives work too well: Locally implemented Pay for performance (P4P) and adverse sanctions towards home birth in Tanzania - a qualitative study. BMC Health Serv Res.

[8] Paul FA. Health worker motivation and the role of performance based financial systems in Africa: A qualitative study on the Rwandan performance based finance initiative in hospitals. *LSE IDS Working Paper Series.* 2009, 44.

[9] Oxman AD, Fretheim A (2009). Can paying for results help to achieve the millennium development goals? overview of the effectiveness of results-based financing. J Evid Based Med.

[10] Mæstad O (2006). Human resources for health in Tanzania: challenges, policy options and knowledge gaps.

[11] Kahabuka C, Kvale G, Moland KM, Hinderaker SG (2011). Why caretakers bypass primary health care facilities for child care - a case from rural Tanzania. BMC Health Serv Res.

[12] Manongi RN, Marchant TC, Bygbjerg C (2006). Improving motivation among primary health care workers in Tanzania: a health worker perspective. Hum Resour Health.

[13] Kruk ME, Rockers PC, Mbaruku G, Paczkowski MM, Galea S (2010). Community and health system factors associated with facility delivery in rural Tanzania: a multilevel analysis. Health Policy.

[14] Larson E, Hermosilla S, Kimweri A, Mbaruku GM, Kruk ME (2014). Determinants of perceived quality of obstetric care in rural Tanzania: a cross-sectional study. BMC Health Serv Res.

[15] Kassebaum NJ, Bertozzi-Villa A, Coggeshall MS, Shackelford KA, Steiner C, Heuton KR, Gonzalez-Medina D, Barber R, Huynh C, Dicker D (2014). Global, regional, and national levels and causes of maternal mortality during 1990–2013: a systematic analysis for the global burden of disease study 2013. Lancet.

[16] UNICEF, World Health Organization, World Bank, United Nations (2014). *Levels & Trends in Child Mortality Report 2014: Estimates Developed by the UN Inter-agency Group for Child Mortality Estimation*.

[17] The United Republic of Tanzania (2011). *The Pwani region pay-for-performance (P4P) pilot: design document*.

[18] Agyepong IA, Adjei S. Public social policy development and implementation: a case study of the Ghana National Health Insurance scheme. *Health Policy and Planning.* 2008;23:1–11. 10.1093/heapol/czn002 18245803

[19] Walt G, Gilson L (1994). Reforming the health sector in developing countries: the central role of policy analysis. Health Policy Plan.

[20] Nyerere JK (1973). Freedom and development : Uhuru na Maendeleo; a selection from writings and speeches 1968–1973.

[21] Pratt C, Julius N (1999). Reflections on the legacy of his socialism. Canadian Journal of African Studies.

[22] Benson JS (2001). The impact of privatization on access in Tanzania. Soc Sci Med.

[23] Holtom D (2005). Reconsidering the Power of the IFIs: Tanzania & the World Bank, 1978–1985. Rev Afr Polit Econ.

[24] Hydén G (2005). *Why do things happen the way they do? A power analysis of Tanzania*.

[25] Rugumamu S (1997). Lethal Aid: the illusion of socialism and self-reliance in Tanzania.

[26] The World Bank: Sub-Saharan Africa (1989). *From crisis to sustainable growth: A long-term perspective study*.

[27] Mkandawire T, Cornwall A, Eade D (2010). Good governance’: the itinerary of an idea. *Deconstructing development discourse: Buzzwords and fuzzwords*.

[28] The World Bank (1994). Governance: The World Bank’s experience.

[29] Helleiner GK, Killick T, Lipumba N, Ndulu BJ, Svendsen SK (1995). Report of the group of independent advisors on development co-operations issues between Tanzania and its Aid donors.

[30] The United Republic of Tanzania (2009). *Health basket fund generic document*.

[31] Crawford G (2003). Partnership or power? deconstructing the ‘partnership for governance reform’ in Indonesia. Third World Q.

[32] Maxwell S, Riddell R (1998). Conditionality or contract: Perspectives on partnership for development. J Int Dev.

[33] Mercer C (2003). Performing partnership: civil society and the illusions of good governance in Tanzania. Polar Geogr Geol.

[34] Green J, Thorogood N (2014). Qualitative methods for health research.

[35] The United Republic of Tanzania (2009). *Health sector strategic plan III (July 2009-June 2015): “Partnership for delivering the MDGs”*.

[36] The United Republic of Tanzania (2008). *The national road Map strategic plan to accelerate reduction of maternal, newborn and child deaths in Tanzania 2008–2015*.

[37] The United Republic of Tanzania (2008). *Payment for performance strategy 2008–2015*.

[38] The United Republic of Tanzania (2008). *Implementation guideline- payment for performance*.

[39] Graneheim UH, Lundman B (2004). Qualitative content analysis in nursing research:concepts, procedures and measures to achieve trustworthiness. Nurse Educ Today.

[40] Oxman AD, A F (2008). *An overview of research on the effects of results-based financing*.

[41] Lauglo M, Swai RBG (2009). *Payment for performance appraisal: Report to norad and the Royal Norwegian Embassy, Tanzania*.

[42] Government plans cash motivation for health workers [http://www.ippmedia.com/frontend/index.php?l=62445]

[43] Stoltenberg J (2006). Our children: the key to our common future. Lancet.

[44] Boseley S (2007). Norway’s Prime Minister Jens Stoltenberg: leader on MDG4. Lancet.

[45] Stoltenberg J (2008). Delivering for women and children. Lancet.

[46] Lomøy J. The Norway-Tanzania Partnership Initiative: A Model for Reducing Child Mortality and Improving Maternal Health. *UN Chronicle.* 2008;45:7–9.

[47] Balkenende JP, Kikwete J, Stoltenberg J, Zoellick R (2008). Innovative finance for women and children. Lancet.

[48] Forstater M, Nakhooda S, Watson C (2013). *The effectiveness of climate finance: a review of the Amazon Fund.* Working Paper 372 edn.

[49] Grindle MS, Thomas JW (1989). Policy makers, policy choices, and policy outcomes: The political economy of reform in developing countries. Policy Sci.

[50] Frenk J, Moon S (2013). Global health governance challenges in global health. New Engl J Med.

[51] Whitefield L, Fraser A (2010). Negotiating Aid: The structural conditions shaping the negotiating strategies of African governments. Int Negot.

[52] Lynge K (2011). Tanzania- the darling of the donor community? a critical review of the failure of past development aid efforts.

