# Supplementary material for: Introducing payment for performance in the health sector of Tanzania- the policy process
Source: Global Health. 2015 Sep 2;11:38. doi: 10.1186/s12992-015-0125-9 (PMC4557903; doi:10.1186/s12992-015-0125-9)
Supplement: Additional files 1: — Interview guide for Norwegian Officials. (DOC 30 kb) [file 12992_2015_125_MOESM1_ESM.doc]

**Interview guide for Norwegian Officials**

**Question 1**

What in your opinion are the main trends or thematic priorities in Norwegian aid policy in recent years, and how would you say the idea of results based aid fits into this landscape?

- Origins of the idea of results-based financing (RBF)
- At what level were the discussions on results-based aid (RBA)
- How was the idea received- e.g political actors, Norad, pressure groups
- Health related-MDGs, relating to MNCH in particular
- With experience in RBA and RBF is opinion changing
- Is funding of RBF shrinking or expanding, in general and in health care
- How has the activities of Norway in the Health Results Innovation Trust Fund provided an impetus into funding P4P initiatives?

**Question 2**

So results based aid has been a part of the Norwegian aid landscape for a while. Where did Norway first get familiar with this way of thinking aid? How long has it been a part of the international aid agenda, and which countries or actors have been its main proponents?

- International players in the RBA and RBF landscape
- Major proponents and opponents of RBA and RBF
- Norway’s role in this landscape
- The role of health related MDGs in promoting P4P
- What other sectors besides health is Norway funding results-based initiatives
- What measures are there to make P4P initiatives sustainable
- What is the role of the financial crisis in promoting results-based aid (RBA)
- What is the role of the Paris Declaration on aid effectiveness in promoting RBA

**Question 3**

The Norwegian- Tanzanian Partnership Initiative (NTPI) which was established in 2007 has been credited for the subsequent introduction of P4P in Tanzania. What has been Norway’s role into this P4P, and what other partners have been influential in this process?

- Origins of the idea of P4P in Tanzania
- What was the response of other development partners
- How come P4P is referred to as the ‘Norwegian thing’
- Who are the prominent actors regarding P4P in Tanzania

**Question 4**

The introduction of P4P in Tanzania has hardly been a smooth process, what experiences has Norway learnt from this process regarding RBF. How has been Norway’s relationship with the government of Tanzania and other development partners when a national P4P was launched in 2009 by authorities in Tanzania?

- Why did it take that long for an agreed approach
- To what extent is Norway in control
- To what extent is the approach (RBF) sustainable
- Does P4P supports earmarking of funds

**Question 5**

Based on the experience from Tanzania has Norway’s belief in P4P as an instrument increased or decreased?

**Other comments**
