# Supplementary material for: Introducing payment for performance in the health sector of Tanzania- the policy process
Source: Global Health. 2015 Sep 2;11:38. doi: 10.1186/s12992-015-0125-9 (PMC4557903; doi:10.1186/s12992-015-0125-9)
Supplement: Additional files 2: — Interview Guide for Tanzanian officials. (DOC 29 kb) [file 12992_2015_125_MOESM2_ESM.doc]

**Interview Guide for Tanzanian officials**

**Question 1**

Tanzania is one of many countries in sub-Saharan Africa currently trying out payment for performance. What were the discussions that lead to the initiating of P4P in Tanzania, and who was involved in these discussions? At what level were these discussions and were they ideological at all?

- Origins of P4P in Tanzania
- Diferent actors involved, political and civic society
- Discourse on MDGs and prioritisation on MNCH
- Discourse on results-based aid in Tanzania
- Perceptions of different actors towards P4P
- State ownership and central planning (Arusha Declaration-Nyerere)
- How do you think P4P will affect the civil service culture in Tanzania

**Question 2**

The health basket is composed of many actors, what are their different roles? Who sets the agenda in the health basket, and who introduced the idea of P4P in the health basket? How does P4P relate to the principles of the health basket?

- What is the role of the MoHSW
- P4P as a ‘Norwegian thing’
- Earmarking of funds/aid
- Role of Norad and CHAI

**Question 3**

In 2009 there were some misunderstandings between Tanzania and its development partners in the health basket regarding the “national P4P roll-out”. What were the major divergent views, and what is your opinion on this matter? What lessons were drawn from this experience and in what ways did this contribute to the shaping of the pilot currently in the Coastal region?

- Were the different views based on ideological or practical issues
- How did the government respond to the reactions of donors
- To what extent does the GoT have ownership of P4P
- Is P4P sustainable in Tanzania
- Does P4P risk damaging other areas of the health sector

**Question 4**

So in 2011 the Pwani region pilot was launched, what has been the experience with this P4P pilot? How does Tanzania benefit from this experience, and what is the opinion in the health baskets on the likelihood of launching a “national P4P”?

- Is the GoT satisfied with how the pilot is going
- What are the results coming from the pilot
- What are the general perceptions surrounding the pilot
- How is the pilot going to be scaled-up
- What are the possibilities of P4P to be implemented in the whole public sector

**Question 5**

Base on your vast experience, in your opinion, do you think implementing P4P is a good idea?

**Other comments**
