# Supplementary material for: Introducing payment for performance in the health sector of Tanzania- the policy process
Source: Global Health. 2015 Sep 2;11:38. doi: 10.1186/s12992-015-0125-9 (PMC4557903; doi:10.1186/s12992-015-0125-9)
Supplement: Additional files 3: — Interview guide for other officials. (DOC 32 kb) [file 12992_2015_125_MOESM3_ESM.doc]

**Interview guide for other officials**

**Question 1**

What in your opinion are the main trends or thematic priorities in development aid policy in recent years, and how would you say the idea of results based aid fits into this landscape?

- Origins of the idea of results-based aid (RBA)
- At what level were the discussions on results-based aid
- How has MDGs in general promoted results-based financing (RBF), and in particular health related MDGs
- What is the role of the financial crisis in promoting results-based aid (RBA)
- What is the role of the Paris Declaration on aid effectiveness in promoting RBA
- With this kind of experience in results-based aid and results-based financing (RBF) is your opinion changing
- How has been the trend in funding RBFs

**Question 2**

Where did your organisation first get familiar with this way of thinking aid? How long has it been a part of the international aid agenda, and which countries or actors have been its main proponents?

- Who are the international players in the RBA and RBF landscape
- Who are the major proponents and opponents of RBA and RBF
- What is your organization’s role in this landscape
- What plans are there on making P4P sustainable

**Question 3**

The health basket is composed of many actors, what are their diferent roles? Who sets the agenda in the health basket, and who introduced the idea of P4P in the health basket? How does P4P relates to the principles of the health basket?

- What is the role of the MoHSW
- What is the role of your organisation
- P4P is referred as a ‘Norwegian thing’ in Tanzania, how do you perceive this way of thinking
- What is your opinion on earmarking of funds/aid, and how do you perceive P4P in relation to earmarking

**Question 4**

The introduction of P4P in Tanzania has hardly been a smooth process, what experiences has your organisation learnt from this process regarding RBFs. How best can you describe the relationship of development partners in the health basket during the discussions, and after introduction of P4P?

**Question 5**

Following the launch of the Pwani region pilot, what has been the experience with this P4P pilot? How is your organisation going to benefit from this experience, and what is the opinion of your organisation on the likelihood of scaling the pilot to national level?

- What are the results coming from the pilot
- What are the general perceptions surrounding the pilot

**Question 6**

Based on your vast experience, in your opinion, is it a good idea to implement P4P?

**Other comments**
